# Crystal structure and characterization of a new lanthanide coordination polymer, [Pr_2_(pydc)(phth)_2_(H_2_O)_3_]·H_2_O

**DOI:** 10.1107/S2056989024000872

**Published:** 2024-01-31

**Authors:** Bunlawee Yotnoi, Apinpus Rujiwatra

**Affiliations:** a Department of Chemistry, School of Science, University of Phayao, Phayao, 56000, Thailand; b Department of Chemistry, Faculty of Science, Chiang Mai University, Chiang Mai, 50200, Thailand; Universidad de Los Andes, Venezuela

**Keywords:** crystal structure, coordination polymer, lanthanide, pyridine-2,5-di­carboxyl­ate, phthalate.

## Abstract

A new lanthanide coordination polymer, poly[[tri­aqua­bis­(μ_4_-phthalato)(μ_3_-pyridine-2,5-di­carboxyl­ato)dipraseodymium] monohydrate], {[Pr_2_(C_7_H_3_NO_4_)_2_(C_8_H_4_O_4_)(H_2_O)_3_]·H_2_O}_*n*_ or {[Pr_2_(phth)_2_(pydc)(H_2_O)_3_]·H_2_O}_*n*_, (pydc^2−^ = pyridine-2,5-di­carboxyl­ate and phth^2−^ = phthalate) was synthesized and characterized, revealing the structure to be an assembly of di-periodic {Pr_2_(pydc)(phth)_2_(H_2_O)_3_}_*n*_ layers. Each layer is built up by edge-sharing {Pr_2_N_2_O_14_} and {Pr_2_O_16_} dimers, which are connected through a new coordin­ation mode of pydc^2−^ and phth^2−^. These layers are stabilized by inter­nal hydrogen bonds and π–π inter­actions. In addition, a three-dimensional supra­molecular framework is built by inter­layer hydrogen-bonding inter­actions involving the non-coordinated water mol­ecule. Thermogravimetric analysis shows that the title compound is thermally stable up to 400°C.

## Abstract 2

The crystal structure and thermogravimetric stability of [Pr_2_(pydc)(phth)_2_(H_2_O)_3_]·H_2_O, a two-dimensional coordination polymer with a novel coordination mode of pyridine-2,5-di­carboxyl­ate (pydc^2−^), are reported.

## 1. Chemical context

Lanthanide coordination polymers (LnCPs) have attracted widespread inter­est because of their unique properties and wide range of potential applications, such as in luminescent temperature sensing (Rocha *et al.*, 2016 ▸), catalysis (Sinchow *et al.*, 2022 ▸), gas detection (Thammakan *et al.*, 2023 ▸) and drug delivery (Wei *et al.*, 2020 ▸). However, the high coordination numbers of the trivalent lanthanides (*Ln* ^III^) and the versatility in their coordination geometries complicates the control of inter­molecular inter­actions and the prediction of coordination polymer frameworks. In addition, the synthesis of these frameworks is also influenced considerably by differences in synthetic procedures and conditions such as solvents, pH, reaction temperature and time, among other factors (Sinchow *et al.*, 2019 ▸). Organic ligands are utilized as a template for the structural design, to direct the framework architecture. Among the organic ligands available, polycarb­oxy­lic acids are notably the most used because they are hard base ligands and can facilitate diverse coordination modes. In this work, pyridine-2,5-di­carb­oxy­lic acid (H_2_pydc) and phthalic acid (H_2_phth) were chosen to be the structure-directing ligands. Relevant structures include, for example, [Pr_3_(phen)_2_(phth)_4_(NO_3_)]·H_2_O (phen = 1,10-phenanthroline) (refcode: LAXWOX; Thirumurugan & Natarajan, 2005 ▸), [Eu(phth)(OAc)(H_2_O)] (OAc = acetate) (refcode: TAZDAD; Jittipiboonwat *et al.*, 2022 ▸), [Pr(pydc)(pip)_1/2_(H_2_O] (pip = 2,5-piperazinedi­carboxyl­ate) (refcode: WUWBIB; Ay *et al.*, 2016 ▸) and [Pr(pydc)(NA)H_2_O]n (NA = nicotinic acid) (refcode: MEJNEY; Hu *et al.*, 2022 ▸). 
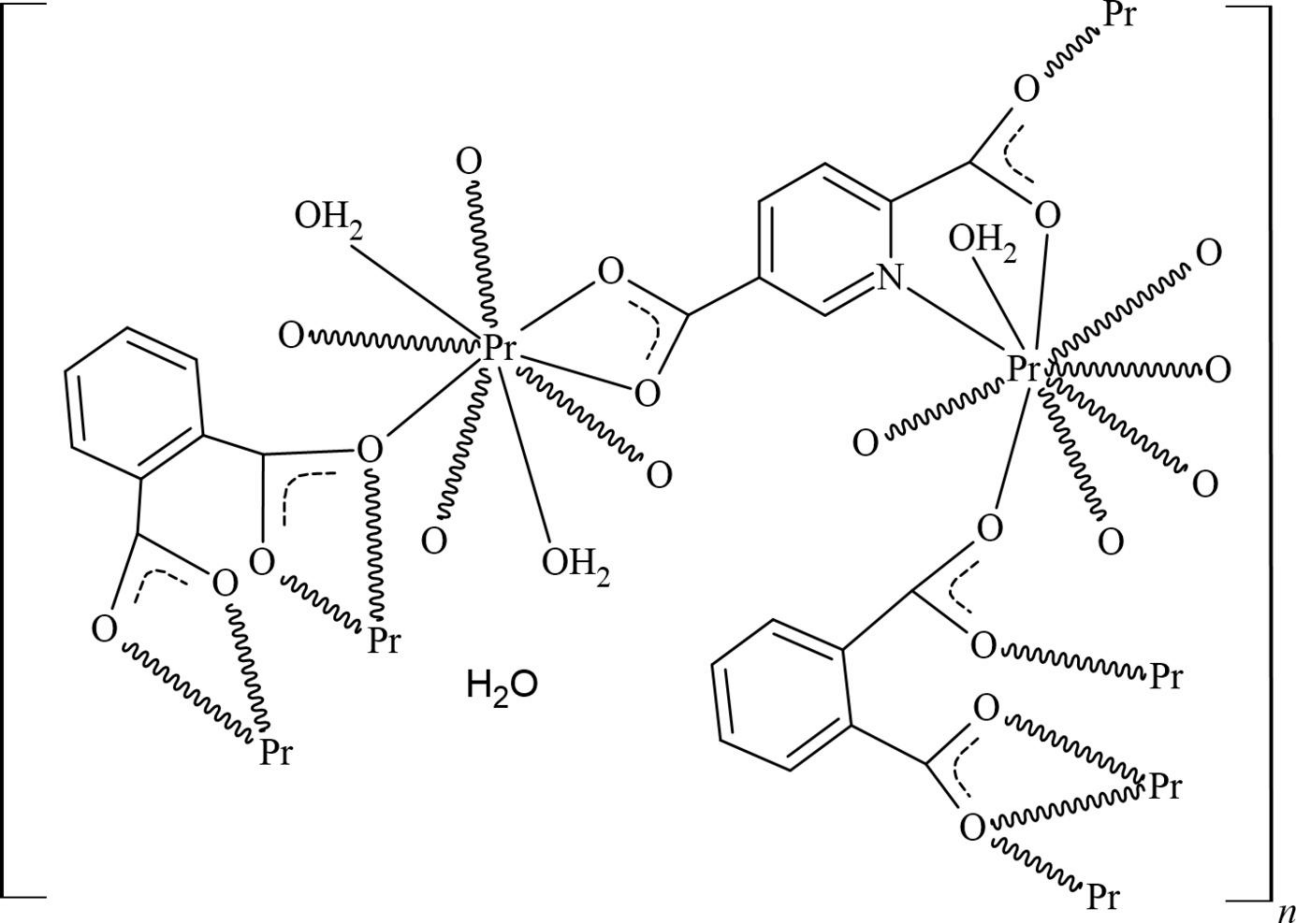


## 2. Structural commentary

**Figure 1:**
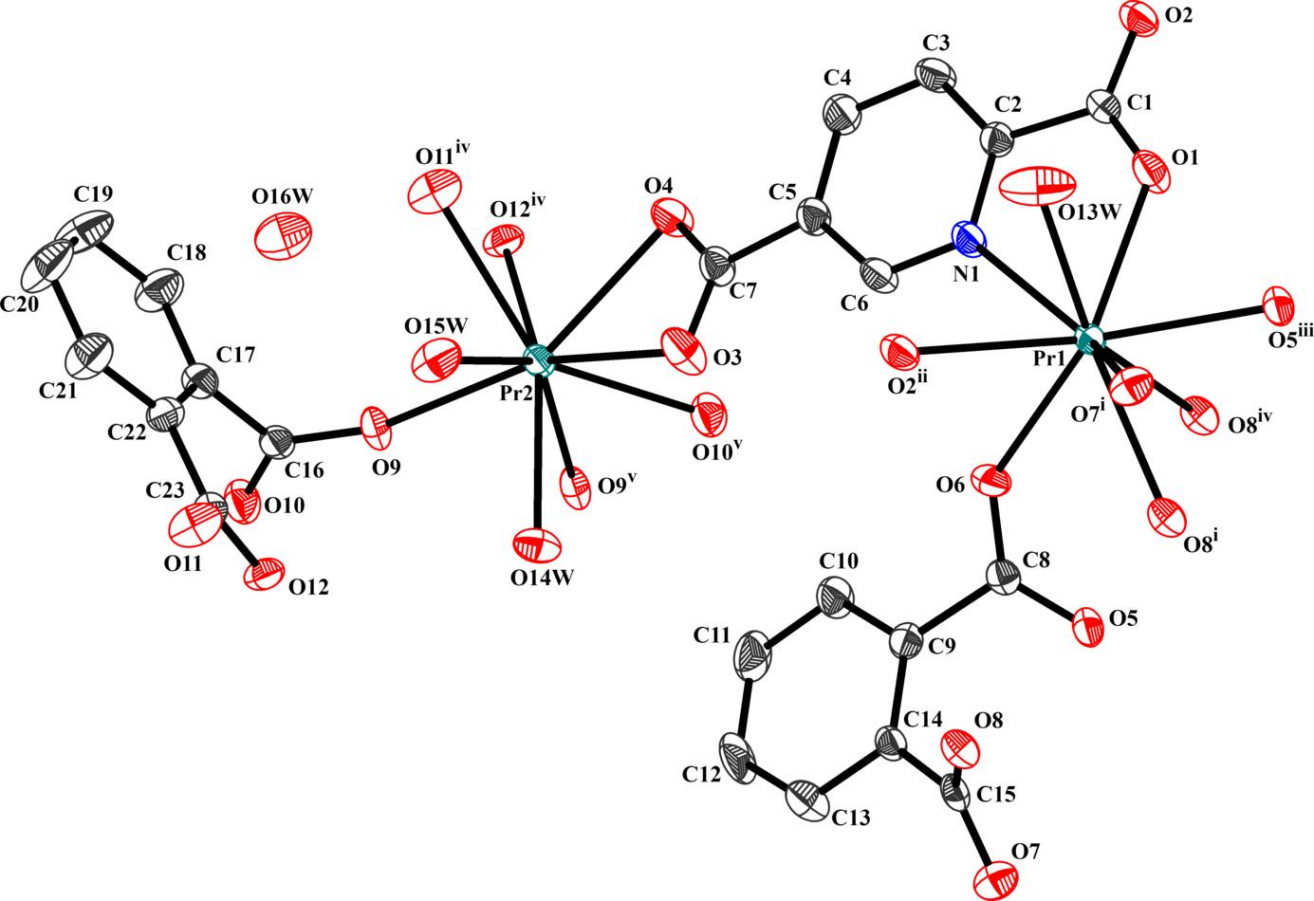
Extended asymmetric unit of [Pr_2_(pydc)(phth)_2_(H_2_O)_3_]·H_2_O drawn using 50% probability for ellipsoids (hydrogen atoms are omitted for clarity). Symmetry codes: (i) −*x* + 1, −*y* + 2, −*z* + 1; (ii) *x*, *y* + 1, *z*; (iii) −*x* + 1, −*y* + 1, −*z* + 1; (iv) *x*, *y* − 1, *z*; (v) −*x* + 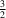 , −*y* + 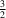 , −*z* + 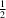 .

**Figure 2:**
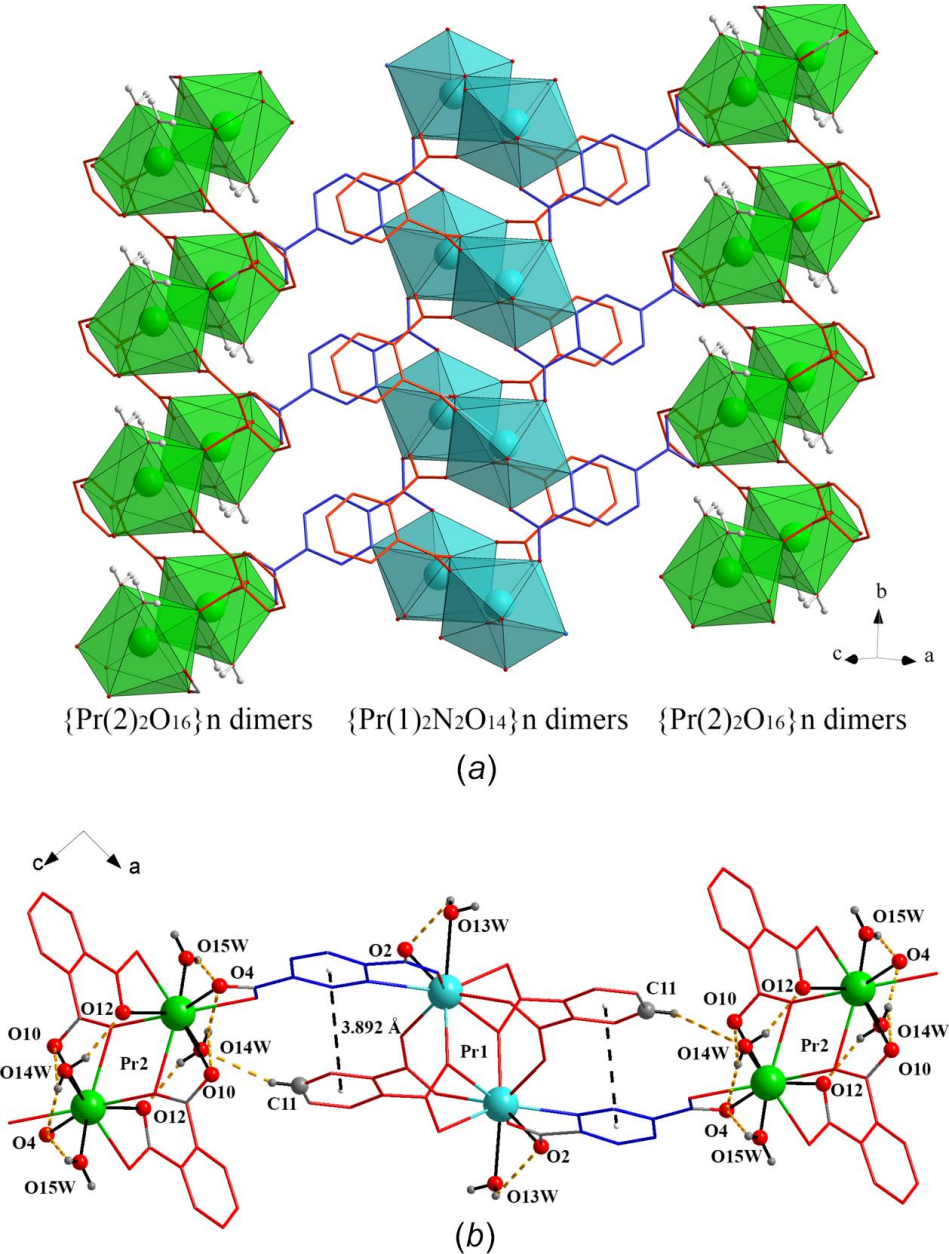
View of (*a*) the {Pr(1)_2_N_2_O_14_} and {Pr(2)_2_O_16_} dimers in the (101) plane and (*b*) the hydrogen bonding and π–π inter­actions in the dimers.

The asymmetric unit of [Pr_2_(pydc)(phth)_2_(H_2_O)_3_]·H_2_O is composed of two Pr^III^ metal centers, one mol­ecule of pydc^2−^, two mol­ecules of phth^2−^, three coordinated water mol­ecules and a non-ligated water mol­ecule (Fig. 1 ▸). The Pr1 ion is ninefold coordinated to one N atom from pydc^2−^ and eight O atoms from four phth^2−^, two pydc^2−^ and one water mol­ecule to form a {Pr(1)NO_8_} motif that can be described as a distorted tricapped trigonal prism. The Pr2 ion is also ninefold coordin­ated, being surrounded by nine O atoms from three phth^2−^, one pydc^2−^ and two water mol­ecules in a distorted tricapped trigonal–prismatic {Pr(2)O_9_} motif. The Pr—O bond lengths are in the range 2.413 (3)–2.691 (3) Å and the Pr—N bond is 2.696 (3) Å (Table 1 ▸), in accordance with a previous report for Pr^III^ frameworks of pydc^2−^ [2.390 (2)–2.717 (3) Å; Sinchow *et al.*, 2019 ▸] and phth^2−^ [2.456 (4)–2.696 (4) Å; Thirumurugan & Natarajan, 2005 ▸]. The {Pr(1)NO_8_} motif is linked to the adjacent Pr1, forming edge-sharing {Pr(1)_2_N_2_O_14_} dimers, and two neighboring {Pr(1)_2_N_2_O_14_} dimers are fused through the *μ*_4_-*η* ^2^:*η* ^1^:*η* ^1^: *η* ^1^ carboxyl group of phth^2−^ to form an infinite chain in the *b*-axis direction. In a similar fashion, two {Pr(2)O_9_} motifs are linked to produce {Pr(2)_2_O_16_} dimers. These dimers are then connected by the carboxyl groups of phth^2−^ in a *μ*_3_-*η* ^2^:*η* ^1^:*η* ^1^:*η* ^1^ fashion to form a mono-periodic chain also extending in the *b*-axis direction. These chains are connected through a novel coordination mode for pydc^2*-*^ involving a *μ*_1_-*η* ^1^:*η* ^1^ carboxyl group at one side and a *μ*_2_-*η* ^1^:*η* ^1^ carboxyl group together with the pyridyl N atom coordinated on the other side to form a {[Pr_2_(pydc)(phth)_2_(H_2_O)_3_]}_*n*_ layer extending in the (101) plane (Fig. 2 ▸ *a*).

## 3. Supra­molecular features

**Figure 3:**
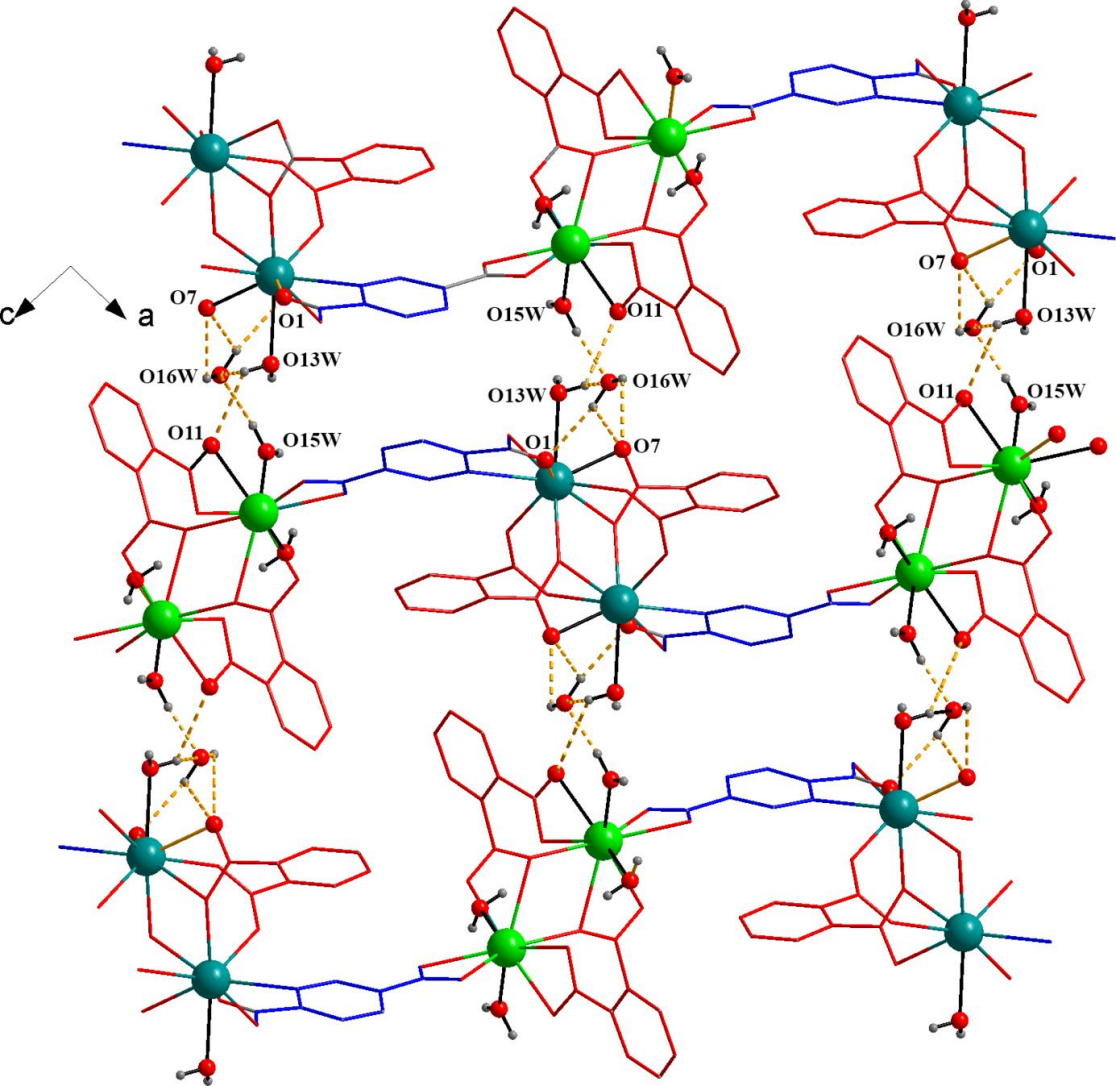
Three-dimensional supra­molecular framework of [Pr_2_(pydc)(phth)_2_(H_2_O)_3_]·H_2_O.

The di-periodic supra­molecular framework of the {[Pr_2_(pydc)(phth)_2_(H_2_O)_3_]}_*n*_ layers is further connected by intra­layer hydrogen bonding, *i.e.* O13*W*—H13*B*⋯O2, O14*W*—H14*A*⋯O12, O14*W*—H14*B*⋯O4, O14*W*—H14*B*⋯O10, O15*W*—H15*B*⋯O4 and C11—H11⋯O14*W* inter­actions and π–π inter­actions (Fig. 2 ▸ *b* and Table 2 ▸). The π–π inter­action between two aromatic rings (pydc^2−^ and phth^2^-) is classified as a parallel stacked geometry (Banerjee *et al.*, 2019 ▸), with an offset of 1.250 Å, inter­planar angle of 5.96° and centroid-to-centroid distance of 3.892 (2) Å. In addition, the inter­layer hydrogen-bonding inter­actions involve the coordin­ated water (O13*W*) and the hydrogen-bonded water (O16*W*). These inter­actions are O13*W*—H13*A*⋯O11, O13*W*—H13*A*⋯O16*W*, O15*W*—H15*A*⋯O16*W*, O16*W*—H16*A*⋯O7, O16*W*—H16*B*⋯O1 and O16*W*—H16*B*⋯O7 inter­actions (Fig. 3 ▸ and Table 2 ▸).

## 4. Thermogravimetric analysis

**Figure 4:**
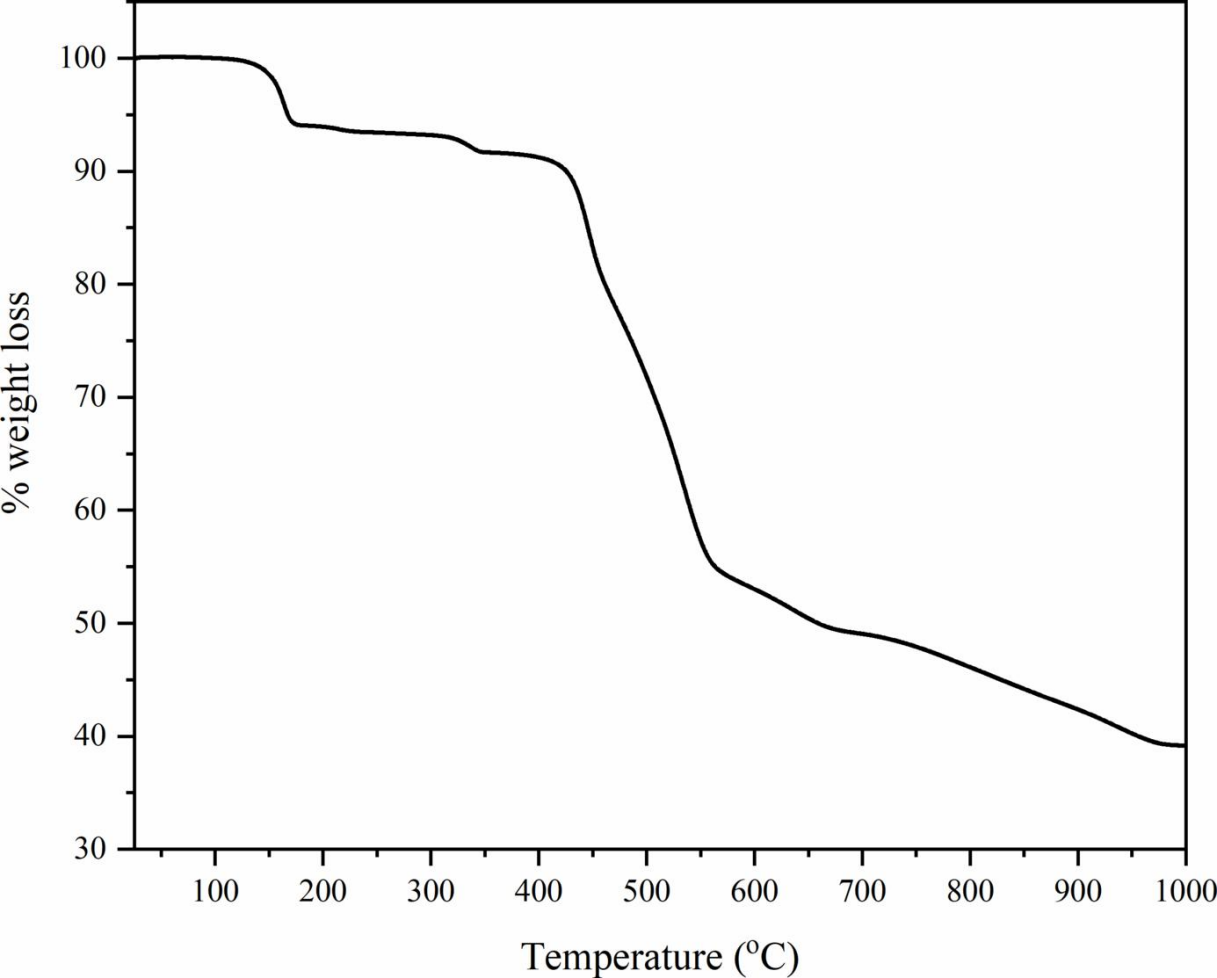
Thermogravimetric analysis of [Pr_2_(pydc)(pth)_2_(H_2_O)_3_]·H_2_O.

The thermogravimetric curve of the title compound shows four steps of weight loss in the temperature range 30°C to 1000°C (Fig. 4 ▸). The first step occurs at 100–185°C with a 6.0% weight loss attributed to the removal of one hydrogen-bonded water and two coordinated water mol­ecules (calc. 6.4%). The second step observed at 300–350°C is due to the loss of the other coordinated water mol­ecule (exp. 2.5%, calc. 2.1%). This step is possibly due to the removal of O14*W*, which is held by both strong and weak hydrogen-bonding inter­actions. The next step of weight loss occurs in the temperature range 400–580°C and represents a higher weight loss of 37.3%. This step can be attributed to the pyrolysis of the organic ligands (two phth^2−^ ligands, calc. 38.7%). The last step of weight loss, from 580 to 1000°C, could be due to the elimination of the bridging pydc^2−^ ligand to form praseodymium oxide residues (exp. 14.7%, calc. 19.5%).

## 5. Database survey

A search for the title compound in the Cambridge Structural Database (CSD version 5.44, April 2023; Groom *et al.*, 2016 ▸) using CONQUEST software (version 2023.2.0; Bruno *et al.*, 2002 ▸) did not match with any reported structures. Regarding organic ligands, there were 123 structures of lanthanide coordination polymers that included pydc^2−^. Among these structures, inter­estingly, there were none in which pydc^2−^ adopts the same coordination mode as in the title compound (Sinchow *et al.*, 2019 ▸). This new mode of coordination acts as a *μ*_3_-bridge to link three Pr^III^ ions and facilitates the formation of a di-periodic coordination framework. Regarding phth^2−^, there were 118 structures deposited in the CSD, none of which contains pydc^2−^ in the structure. However, there is a structure including both pydc^2−^ and phth^2−^ ligands that incorporates a first-row transition metal: [Gd_2_(H_2_O)_2_Ni(H_2_O)_2_(phth)_2_(pydc)_2_]_3_·8H_2_O (refcode: XOZYER; Mahata *et al.*, 2009 ▸).

## 6. Synthesis and crystallization

All chemicals were used as received without further purification: Pr_6_O_11_ (TJTM, 99.9%), pyridine-2,5-di­carb­oxy­lic acid (H_2_pydc; Sigma-Aldrich, 98%), 1,2-benzene­dicarb­oxy­lic acid (H_2_phth; Sigma-Aldrich, 98%) and NaOH (QReC, 99%). The Pr(NO_3_)_3_·6H_2_O precursor was prepared by crystallization from solution of the lanthanide oxide in nitric acid (RCI Labscan, 65%).

To synthesize [Pr_2_(pydc)(phth)_2_(H_2_O)_3_]·H_2_O, a solution of H_2_pydc (0.125 mmol, 20.8 mg) and H_2_phth (0.25 mmol, 41.5 mg) was prepared in 8 mL of deionized water, then 1.35 mL of 0.5 *M* NaOH were added and the pH adjusted to 5. Pr(NO_3_)·6H_2_O (0.25 mmol, 146.6 mg) was dissolved in 2 mL of deionized water and mixed with the ligand solution. The reaction mixture was then transferred into a 23 mL Teflon-lined hydro­thermal reactor and held at 423 K for 72 h. Green block-shaped crystals were collected and dried at room temperature. The crystals were characterized using FT–IR spectroscopy (Nicolet iS5 FTIR Spectrometer; iD5 ATR mode; cm^−1^): 3243(*br*), 1615(*w*), 1575(*m*), 1543(*m*), 1517(*m*), 1481(*m*), 1450(*w*), 1391(*m*), 1354(*m*), 1283(*w*), 1143(*w*), 1088(*w*), 1028(*w*), 870(*w*), 840(*m*), 762(*m*), 670(*m*), 648(*w*). The FT–IR spectrum shows a broad band at 3243 cm^−1^ attributed to the *ν*(O—H) stretching from the water mol­ecules. The characteristic peak at 1615 cm^−1^ corresponds to the C=O stretching vibrational mode of the carboxyl­ate group. The peak at 1283 cm^−1^ is due to the C—N stretching of the pydc^2−^ ligand.

Thermogravimetric analyses (TGA) were carried out using a Mettler Toledo TGA/DSC 3+, with a heating rate of 20°C min^−1^, ramping from 30 to 1100°C under a nitro­gen gas flow.

## 7. Refinement

Crystal data, data collection and structure refinement details are summarized in Table 3 ▸. All hydrogen atoms of aromatic rings and water mol­ecules were positioned geometrically and refined using a riding model with *U_i_*_so_(H) = 1.2–1.5*U*_eq_(C,O).

## Supplementary Material

Crystal structure: contains datablock(s) I. DOI: 10.1107/S2056989024000872/jw2002sup1.cif

Structure factors: contains datablock(s) I. DOI: 10.1107/S2056989024000872/jw2002Isup3.hkl

CCDC reference: 2327873

Additional supporting information:  crystallographic information; 3D view; checkCIF report

## Figures and Tables

**Table 1:** Selected bond lengths (Å)

Pr1—O7^i^	2.536 (3)	Pr2—O12^iv^	2.514 (2)
Pr1—O2^ii^	2.580 (2)	Pr2—O15*W*	2.413 (3)
Pr1—O5^iii^	2.473 (2)	Pr2—O9^v^	2.691 (3)
Pr1—O8^i^	2.689 (2)	Pr2—O9	2.469 (2)
Pr1—O8^iv^	2.473 (3)	Pr2—O4	2.526 (2)
Pr1—O6	2.446 (3)	Pr2—O10^v^	2.510 (3)
Pr1—O1	2.439 (2)	Pr2—O3	2.575 (3)
Pr1—N1	2.696 (3)	Pr2—O14*W*	2.460 (2)
Pr1—O13*W*	2.571 (4)	Pr2—O11^iv^	2.529 (3)

Symmetry codes: (i) 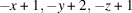 ; (ii) 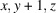 ; (iii) 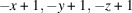 ; (iv) 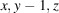 ; (v) 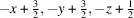 .

**Table 2:** Hydrogen-bond geometry (Å, °)

*D*—H⋯*A*	*D*—H	H⋯*A*	*D*⋯*A*	*D*—H⋯*A*
O13*W*—H13*A*⋯O11^vi^	0.85	2.57	3.003 (5)	113
O13*W*—H13*A*⋯O16*W* ^vi^	0.85	2.26	2.881 (5)	130
O13*W*—H13*B*⋯O2^ii^	0.86	2.52	2.820 (4)	102
O14*W*—H14*A*⋯O12^vii^	0.85	1.95	2.766 (4)	160
O14*W*—H14*B*⋯O4^ii^	0.85	2.04	2.887 (4)	174
O14*W*—H14*B*⋯O10^vii^	0.85	2.58	2.912 (4)	104
O15*W*—H15*A*⋯O16*W*	0.85	1.75	2.595 (4)	170
O15*W*—H15*B*⋯O4^ii^	0.85	1.92	2.725 (3)	158
O16*W*—H16*B*⋯O7^viii^	0.85	2.31	2.709 (4)	109
O16*W*—H16*B*⋯O1^ix^	0.85	2.13	2.938 (5)	158
C11—H11⋯O14*W*	0.93	2.51	3.375 (5)	156

Symmetry codes: (ii) 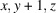 ; (vi) 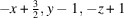 ; (vii) 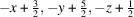 ; (viii) 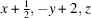 ; (ix) 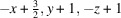 .

**Table 3:** Experimental details

Crystal data	
Chemical formula	[Pr_2_(C_7_H_3_NO_4_)_2_(C_8_H_4_O_4_)(H_2_O)_3_]·H_2_O
*M*_r_	847.21
Crystal system, space group	Monoclinic, *I*2/*a*
Temperature (K)	293
*a*, *b*, *c* (Å)	27.4898 (4), 5.9436 (1), 32.0473 (5)
β (°)	93.854 (1)
*V* (Å^3^)	5224.31 (14)
*Z*	8
Radiation type	Mo *K*α
μ (mm^−1^)	3.77
Crystal size (mm)	0.3 × 0.2 × 0.08

Data collection	
Diffractometer	SuperNova, Single source at offset/far, HyPix3000
Absorption correction	Multi-scan (*CrysAlis PRO*; Rigaku OD, 2019 ▸)
*T*_min_, *T*_max_	0.448, 1.000
No. of measured, independent and observed [*I* > 2σ(*I*)] reflections	26747, 5561, 5004
*R*_int_	0.062
(sin θ/λ)_max_ (Å^−1^)	0.648

Refinement	
*R*[*F* ^2^ > 2σ(*F* ^2^)], *wR*(*F* ^2^), *S*	0.031, 0.081, 1.05
No. of reflections	5561
No. of parameters	387
H-atom treatment	H-atom parameters constrained
Δρ_max_, Δρ_min_ (e Å^−3^)	1.03, −1.08

Computer programs: *CrysAlis PRO* (Rigaku OD, 2019 ▸), *SHELXT2018/2* (Sheldrick, 2015*a* ▸), *SHELXL2018/3* (Sheldrick, 2015*b* ▸) and *OLEX2* (Dolomanov *et al.*, 2009 ▸).

## supplementary crystallographic information

### Crystal data

**Table:**

[Pr_2_(C_7_H_3_NO_4_)_2_(C_8_H_4_O_4_)(H_2_O)_3_]·H_2_O	*F*(000) = 3280
*M**_r_* = 847.21	*D*_x_ = 2.154 Mg m^−^^3^
Monoclinic, *I*2/*a*	Mo *K*α radiation, λ = 0.71073 Å
*a* = 27.4898 (4) Å	Cell parameters from 20454 reflections
*b* = 5.9436 (1) Å	θ = 2.0–27.3°
*c* = 32.0473 (5) Å	µ = 3.77 mm^−^^1^
β = 93.854 (1)°	*T* = 293 K
*V* = 5224.31 (14) Å^3^	Block, clear light green
*Z* = 8	0.3 × 0.2 × 0.08 mm

### Data collection

**Table:**

SuperNova, Single source at offset/far, HyPix3000 diffractometer	5561 independent reflections
Radiation source: micro-focus sealed X-ray tube, SuperNova (Mo) X-ray Source	5004 reflections with *I* > 2σ(*I*)
Mirror monochromator	*R*_int_ = 0.062
Detector resolution: 10.0000 pixels mm^-1^	θ_max_ = 27.4°, θ_min_ = 1.9°
ω scans	*h* = −32→34
Absorption correction: multi-scan (CrysAlisPro; Rigaku OD, 2019)	*k* = −7→7
*T*_min_ = 0.448, *T*_max_ = 1.000	*l* = −40→40
26747 measured reflections	

### Refinement

**Table:**

Refinement on *F*^2^	Primary atom site location: dual
Least-squares matrix: full	Hydrogen site location: mixed
*R*[*F*^2^ > 2σ(*F*^2^)] = 0.031	H-atom parameters constrained
*wR*(*F*^2^) = 0.081	*w* = 1/[σ^2^(*F*_o_^2^) + (0.0445*P*)^2^] where *P* = (*F*_o_^2^ + 2*F*_c_^2^)/3
*S* = 1.05	(Δ/σ)_max_ = 0.003
5561 reflections	Δρ_max_ = 1.03 e Å^−^^3^
387 parameters	Δρ_min_ = −1.08 e Å^−^^3^
0 restraints	

### Special details

**Table:**

Geometry. All esds (except the esd in the dihedral angle between two l.s. planes) are estimated using the full covariance matrix. The cell esds are taken into account individually in the estimation of esds in distances, angles and torsion angles; correlations between esds in cell parameters are only used when they are defined by crystal symmetry. An approximate (isotropic) treatment of cell esds is used for estimating esds involving l.s. planes.
Refinement. The crystal structure was solved using the dual-space algorithm with the SHELXT program (Sheldrick, 2015a) and refined on F^2^ by the full-matrix least-squares technique using the SHELXL program (Sheldrick, 2015b) via the Olex2 interface (Dolomanov *et al.*, 2009).

### Fractional atomic coordinates and isotropic or equivalent isotropic displacement parameters (Å^2^)

**Table:**

	*x*	*y*	*z*	*U*_iso_*/*U*_eq_	
Pr1	0.57085 (2)	0.43694 (3)	0.52003 (2)	0.01775 (8)	
Pr2	0.75715 (2)	0.65833 (3)	0.31515 (2)	0.01807 (8)	
O7	0.45283 (10)	1.3428 (4)	0.41664 (9)	0.0278 (6)	
O2	0.63896 (9)	−0.2639 (4)	0.52143 (8)	0.0258 (6)	
O12	0.80011 (9)	1.3574 (4)	0.27660 (8)	0.0233 (6)	
O5	0.48823 (9)	0.7809 (4)	0.44133 (8)	0.0245 (6)	
O8	0.50558 (9)	1.2895 (4)	0.47057 (8)	0.0225 (6)	
O15W	0.79900 (10)	0.9333 (4)	0.35981 (10)	0.0348 (7)	
H15A	0.828906	0.931811	0.368452	0.052*	
H15B	0.788865	1.062225	0.366759	0.052*	
O9	0.79607 (10)	0.8562 (4)	0.25842 (8)	0.0247 (6)	
O4	0.74925 (10)	0.3284 (4)	0.36394 (9)	0.0288 (7)	
O10	0.82266 (10)	1.0318 (4)	0.20438 (9)	0.0285 (6)	
O6	0.56535 (10)	0.6744 (4)	0.45754 (9)	0.0270 (6)	
O3	0.71029 (11)	0.6308 (4)	0.38190 (9)	0.0332 (7)	
O14W	0.70712 (9)	1.0001 (4)	0.30575 (9)	0.0272 (6)	
H14A	0.711517	1.058376	0.282044	0.041*	
H14B	0.717297	1.099306	0.323394	0.041*	
O11	0.84297 (10)	1.5114 (5)	0.32951 (9)	0.0328 (7)	
O1	0.59351 (10)	0.0418 (4)	0.52815 (9)	0.0314 (7)	
N1	0.63969 (11)	0.2702 (5)	0.47251 (10)	0.0237 (7)	
O16W	0.89141 (13)	0.9700 (6)	0.38119 (11)	0.0511 (9)	
H16A	0.898260	0.843887	0.370560	0.077*	
H16B	0.898930	0.954362	0.407190	0.077*	
O13W	0.63638 (14)	0.3845 (6)	0.58034 (11)	0.0575 (10)	
H13A	0.623288	0.331315	0.601685	0.086*	
H13B	0.647446	0.512964	0.588387	0.086*	
C15	0.49303 (14)	1.2686 (5)	0.43171 (12)	0.0201 (8)	
C22	0.88149 (13)	1.2236 (6)	0.29160 (12)	0.0224 (8)	
C17	0.87562 (14)	1.0344 (6)	0.26623 (12)	0.0226 (8)	
C23	0.83927 (13)	1.3731 (6)	0.29984 (12)	0.0214 (8)	
C6	0.66161 (14)	0.3836 (6)	0.44322 (12)	0.0236 (8)	
H6	0.650321	0.527214	0.436258	0.028*	
C16	0.82826 (14)	0.9752 (6)	0.24196 (12)	0.0209 (8)	
C2	0.65666 (13)	0.0621 (5)	0.48235 (12)	0.0200 (8)	
C8	0.53344 (13)	0.7948 (5)	0.43889 (12)	0.0192 (8)	
C5	0.70056 (13)	0.2984 (6)	0.42248 (12)	0.0229 (8)	
C1	0.62796 (14)	−0.0633 (5)	0.51323 (12)	0.0196 (8)	
C14	0.52822 (13)	1.1759 (5)	0.40244 (12)	0.0194 (8)	
C7	0.72085 (14)	0.4317 (6)	0.38760 (12)	0.0236 (9)	
C18	0.91496 (15)	0.8966 (7)	0.26096 (15)	0.0364 (11)	
H18	0.911010	0.769656	0.244119	0.044*	
C21	0.92728 (15)	1.2708 (7)	0.31040 (14)	0.0347 (10)	
H21	0.931630	1.397619	0.327226	0.042*	
C4	0.71830 (15)	0.0895 (6)	0.43396 (13)	0.0299 (10)	
H4	0.744967	0.029575	0.421450	0.036*	
C11	0.59980 (15)	1.0454 (7)	0.35039 (14)	0.0355 (11)	
H11	0.623695	1.001566	0.332856	0.043*	
C3	0.69586 (14)	−0.0308 (6)	0.46450 (12)	0.0266 (9)	
H3	0.707294	−0.172427	0.472697	0.032*	
C10	0.58662 (14)	0.9040 (6)	0.38164 (13)	0.0271 (9)	
H10	0.602064	0.765556	0.385436	0.033*	
C9	0.55037 (13)	0.9663 (6)	0.40764 (12)	0.0213 (8)	
C13	0.54177 (15)	1.3151 (6)	0.37105 (13)	0.0300 (10)	
H13	0.526545	1.453984	0.367252	0.036*	
C12	0.57745 (16)	1.2535 (7)	0.34501 (14)	0.0377 (11)	
H12	0.586413	1.350458	0.324088	0.045*	
C19	0.96019 (16)	0.9438 (8)	0.28028 (16)	0.0454 (13)	
H19	0.986346	0.847910	0.276856	0.054*	
C20	0.96632 (17)	1.1326 (8)	0.30449 (17)	0.0485 (13)	
H20	0.996917	1.167448	0.316952	0.058*	

### Atomic displacement parameters (Å^2^)

**Table:**

	*U*^11^	*U*^22^	*U*^33^	*U*^12^	*U*^13^	*U*^23^
Pr1	0.01815 (13)	0.01481 (12)	0.02073 (14)	0.00126 (7)	0.00452 (9)	0.00299 (8)
Pr2	0.01974 (13)	0.01567 (12)	0.01921 (14)	0.00040 (7)	0.00422 (9)	0.00159 (8)
O7	0.0247 (15)	0.0284 (14)	0.0302 (17)	0.0052 (12)	0.0009 (13)	−0.0047 (12)
O2	0.0305 (15)	0.0164 (12)	0.0310 (16)	0.0006 (11)	0.0054 (13)	0.0040 (11)
O12	0.0186 (14)	0.0230 (13)	0.0278 (16)	0.0040 (11)	−0.0032 (12)	0.0000 (11)
O5	0.0222 (14)	0.0220 (13)	0.0300 (16)	−0.0022 (11)	0.0078 (12)	0.0052 (12)
O8	0.0312 (15)	0.0179 (12)	0.0184 (15)	−0.0023 (11)	0.0024 (12)	−0.0003 (11)
O15W	0.0267 (16)	0.0304 (15)	0.046 (2)	0.0023 (12)	−0.0064 (15)	−0.0147 (14)
O9	0.0262 (15)	0.0207 (12)	0.0280 (16)	−0.0066 (11)	0.0077 (12)	0.0032 (11)
O4	0.0362 (17)	0.0229 (13)	0.0292 (17)	0.0071 (12)	0.0155 (14)	0.0060 (11)
O10	0.0278 (15)	0.0332 (15)	0.0245 (16)	−0.0063 (12)	0.0020 (12)	0.0058 (12)
O6	0.0262 (15)	0.0261 (14)	0.0288 (16)	0.0055 (11)	0.0032 (13)	0.0105 (12)
O3	0.052 (2)	0.0198 (13)	0.0305 (17)	0.0036 (13)	0.0216 (15)	0.0004 (12)
O14W	0.0332 (16)	0.0228 (13)	0.0256 (16)	0.0057 (12)	0.0024 (13)	0.0019 (11)
O11	0.0314 (16)	0.0359 (15)	0.0299 (17)	0.0070 (13)	−0.0053 (13)	−0.0145 (13)
O1	0.0379 (17)	0.0202 (13)	0.0386 (18)	0.0036 (12)	0.0206 (14)	0.0074 (12)
N1	0.0259 (17)	0.0156 (14)	0.0306 (19)	0.0024 (13)	0.0097 (15)	0.0037 (14)
O16W	0.046 (2)	0.059 (2)	0.046 (2)	0.0062 (18)	−0.0142 (18)	0.0038 (17)
O13W	0.072 (3)	0.054 (2)	0.044 (2)	0.0244 (19)	−0.0162 (19)	0.0018 (17)
C15	0.023 (2)	0.0133 (16)	0.024 (2)	−0.0063 (15)	0.0044 (17)	0.0010 (15)
C22	0.021 (2)	0.0225 (18)	0.023 (2)	0.0021 (16)	0.0008 (16)	0.0015 (16)
C17	0.023 (2)	0.0228 (18)	0.022 (2)	0.0010 (16)	0.0028 (16)	0.0041 (16)
C23	0.020 (2)	0.0191 (17)	0.025 (2)	0.0008 (15)	0.0029 (17)	0.0022 (16)
C6	0.030 (2)	0.0174 (16)	0.024 (2)	0.0029 (16)	0.0075 (18)	0.0038 (16)
C16	0.021 (2)	0.0173 (17)	0.025 (2)	0.0039 (15)	0.0078 (17)	0.0030 (16)
C2	0.020 (2)	0.0177 (17)	0.022 (2)	0.0009 (14)	0.0037 (16)	−0.0003 (15)
C8	0.022 (2)	0.0146 (16)	0.021 (2)	0.0011 (15)	0.0046 (16)	−0.0022 (15)
C5	0.024 (2)	0.0221 (18)	0.023 (2)	0.0001 (16)	0.0077 (17)	0.0028 (16)
C1	0.023 (2)	0.0170 (17)	0.019 (2)	−0.0009 (15)	0.0020 (16)	0.0020 (14)
C14	0.0198 (19)	0.0174 (17)	0.021 (2)	−0.0037 (14)	0.0003 (16)	0.0019 (14)
C7	0.028 (2)	0.0210 (19)	0.023 (2)	−0.0004 (16)	0.0091 (18)	0.0012 (16)
C18	0.027 (2)	0.035 (2)	0.047 (3)	0.0083 (19)	0.004 (2)	−0.007 (2)
C21	0.026 (2)	0.034 (2)	0.044 (3)	0.0017 (19)	−0.001 (2)	−0.009 (2)
C4	0.029 (2)	0.0241 (19)	0.039 (3)	0.0037 (17)	0.014 (2)	0.0032 (18)
C11	0.029 (2)	0.046 (3)	0.033 (3)	−0.0025 (19)	0.015 (2)	0.003 (2)
C3	0.032 (2)	0.0202 (18)	0.029 (2)	0.0065 (16)	0.0095 (18)	0.0060 (16)
C10	0.028 (2)	0.0261 (19)	0.028 (2)	0.0016 (17)	0.0112 (18)	0.0018 (17)
C9	0.0182 (19)	0.0225 (18)	0.024 (2)	−0.0012 (15)	0.0035 (16)	0.0002 (16)
C13	0.039 (3)	0.0239 (19)	0.027 (2)	0.0015 (17)	0.005 (2)	0.0052 (17)
C12	0.043 (3)	0.039 (2)	0.033 (3)	−0.006 (2)	0.018 (2)	0.014 (2)
C19	0.026 (2)	0.053 (3)	0.056 (3)	0.018 (2)	0.000 (2)	−0.010 (3)
C20	0.018 (2)	0.063 (3)	0.063 (4)	0.002 (2)	−0.008 (2)	−0.012 (3)

### Geometric parameters (Å, º)

**Table:**

Pr1—Pr1^i^	4.0877 (4)	N1—C2	1.352 (4)
Pr1—O7^ii^	2.536 (3)	O16W—H16A	0.8495
Pr1—O2^iii^	2.580 (2)	O16W—H16B	0.8500
Pr1—O5^i^	2.473 (2)	O13W—H13A	0.8548
Pr1—O8^ii^	2.689 (2)	O13W—H13B	0.8552
Pr1—O8^iv^	2.473 (3)	C15—C14	1.497 (5)
Pr1—O6	2.446 (3)	C22—C17	1.391 (5)
Pr1—O1	2.439 (2)	C22—C23	1.499 (5)
Pr1—N1	2.696 (3)	C22—C21	1.387 (5)
Pr1—O13W	2.571 (4)	C17—C16	1.513 (5)
Pr1—C15^ii^	2.984 (4)	C17—C18	1.376 (5)
Pr2—O12^iv^	2.514 (2)	C6—H6	0.9300
Pr2—O15W	2.413 (3)	C6—C5	1.393 (5)
Pr2—O9^v^	2.691 (3)	C2—C1	1.504 (5)
Pr2—O9	2.469 (2)	C2—C3	1.370 (5)
Pr2—O4	2.526 (2)	C8—C9	1.523 (5)
Pr2—O10^v^	2.510 (3)	C5—C7	1.507 (5)
Pr2—O3	2.575 (3)	C5—C4	1.375 (5)
Pr2—O14W	2.460 (2)	C14—C9	1.392 (5)
Pr2—O11^iv^	2.529 (3)	C14—C13	1.373 (5)
Pr2—C23^iv^	2.891 (4)	C18—H18	0.9300
Pr2—C16^v^	2.986 (4)	C18—C19	1.380 (6)
O7—C15	1.256 (5)	C21—H21	0.9300
O2—C1	1.254 (4)	C21—C20	1.374 (6)
O12—C23	1.271 (4)	C4—H4	0.9300
O5—C8	1.253 (4)	C4—C3	1.390 (5)
O8—C15	1.276 (4)	C11—H11	0.9300
O15W—H15A	0.8498	C11—C10	1.375 (5)
O15W—H15B	0.8499	C11—C12	1.386 (5)
O9—C16	1.274 (4)	C3—H3	0.9300
O4—C7	1.281 (4)	C10—H10	0.9300
O10—C16	1.250 (4)	C10—C9	1.391 (5)
O6—C8	1.252 (4)	C13—H13	0.9300
O3—C7	1.229 (4)	C13—C12	1.379 (5)
O14W—H14A	0.8511	C12—H12	0.9300
O14W—H14B	0.8512	C19—H19	0.9300
O11—C23	1.256 (4)	C19—C20	1.368 (6)
O1—C1	1.255 (4)	C20—H20	0.9300
N1—C6	1.332 (4)		
			
O7^ii^—Pr1—Pr1^i^	82.31 (6)	C23^iv^—Pr2—C16^v^	109.32 (10)
O7^ii^—Pr1—O2^iii^	81.68 (8)	C15—O7—Pr1^ii^	98.1 (2)
O7^ii^—Pr1—O8^ii^	49.76 (8)	C1—O2—Pr1^iv^	119.2 (2)
O7^ii^—Pr1—N1	150.37 (10)	C23—O12—Pr2^iii^	93.8 (2)
O7^ii^—Pr1—O13W	70.46 (10)	C8—O5—Pr1^i^	138.9 (2)
O7^ii^—Pr1—C15^ii^	24.63 (10)	Pr1^iii^—O8—Pr1^ii^	104.62 (8)
O2^iii^—Pr1—Pr1^i^	123.65 (6)	C15—O8—Pr1^iii^	142.8 (2)
O2^iii^—Pr1—O8^ii^	98.79 (7)	C15—O8—Pr1^ii^	90.4 (2)
O2^iii^—Pr1—N1	74.17 (8)	Pr2—O15W—H15A	126.8
O2^iii^—Pr1—C15^ii^	92.20 (8)	Pr2—O15W—H15B	127.9
O5^i^—Pr1—Pr1^i^	67.00 (6)	H15A—O15W—H15B	104.5
O5^i^—Pr1—O7^ii^	69.95 (8)	Pr2—O9—Pr2^v^	113.13 (9)
O5^i^—Pr1—O2^iii^	148.44 (9)	C16—O9—Pr2	156.4 (3)
O5^i^—Pr1—O8^ii^	73.52 (7)	C16—O9—Pr2^v^	90.4 (2)
O5^i^—Pr1—N1	126.85 (8)	C7—O4—Pr2	94.3 (2)
O5^i^—Pr1—O13W	90.61 (11)	C16—O10—Pr2^v^	99.7 (2)
O5^i^—Pr1—C15^ii^	67.57 (8)	C8—O6—Pr1	136.3 (2)
O8^iv^—Pr1—Pr1^i^	39.54 (6)	C7—O3—Pr2	93.4 (2)
O8^ii^—Pr1—Pr1^i^	35.83 (6)	Pr2—O14W—H14A	109.5
O8^iv^—Pr1—O7^ii^	118.78 (9)	Pr2—O14W—H14B	109.6
O8^iv^—Pr1—O2^iii^	138.56 (8)	H14A—O14W—H14B	104.5
O8^iv^—Pr1—O5^i^	70.29 (9)	C23—O11—Pr2^iii^	93.5 (2)
O8^iv^—Pr1—O8^ii^	75.37 (8)	C1—O1—Pr1	129.2 (2)
O8^ii^—Pr1—N1	150.31 (9)	C6—N1—Pr1	125.4 (2)
O8^iv^—Pr1—N1	90.82 (9)	C6—N1—C2	117.5 (3)
O8^iv^—Pr1—O13W	151.68 (10)	C2—N1—Pr1	116.7 (2)
O8^iv^—Pr1—C15^ii^	96.50 (10)	H16A—O16W—H16B	104.5
O8^ii^—Pr1—C15^ii^	25.31 (9)	Pr1—O13W—H13A	109.6
O6—Pr1—Pr1^i^	68.41 (6)	Pr1—O13W—H13B	109.6
O6—Pr1—O7^ii^	110.62 (9)	H13A—O13W—H13B	104.4
O6—Pr1—O2^iii^	67.76 (9)	O7—C15—Pr1^ii^	57.32 (19)
O6—Pr1—O5^i^	134.86 (9)	O7—C15—O8	121.0 (3)
O6—Pr1—O8^iv^	71.19 (9)	O7—C15—C14	118.5 (3)
O6—Pr1—O8^ii^	74.76 (8)	O8—C15—Pr1^ii^	64.34 (18)
O6—Pr1—N1	75.93 (9)	O8—C15—C14	120.2 (3)
O6—Pr1—O13W	133.39 (12)	C14—C15—Pr1^ii^	165.5 (2)
O6—Pr1—C15^ii^	94.57 (9)	C17—C22—C23	121.4 (3)
O1—Pr1—Pr1^i^	116.18 (7)	C21—C22—C17	118.9 (3)
O1—Pr1—O7^ii^	119.27 (9)	C21—C22—C23	119.6 (3)
O1—Pr1—O2^iii^	118.80 (9)	C22—C17—C16	123.4 (3)
O1—Pr1—O5^i^	67.30 (8)	C18—C17—C22	119.5 (4)
O1—Pr1—O8^ii^	140.00 (8)	C18—C17—C16	117.1 (3)
O1—Pr1—O8^iv^	84.05 (9)	O12—C23—Pr2^iii^	60.15 (18)
O1—Pr1—O6	130.10 (9)	O12—C23—C22	119.2 (3)
O1—Pr1—N1	61.47 (8)	O11—C23—Pr2^iii^	60.80 (19)
O1—Pr1—O13W	69.03 (11)	O11—C23—O12	121.0 (3)
O1—Pr1—C15^ii^	131.64 (9)	O11—C23—C22	119.8 (4)
N1—Pr1—Pr1^i^	125.47 (7)	C22—C23—Pr2^iii^	179.4 (3)
N1—Pr1—C15^ii^	165.50 (9)	N1—C6—H6	118.3
O13W—Pr1—Pr1^i^	149.66 (8)	N1—C6—C5	123.4 (3)
O13W—Pr1—O2^iii^	66.39 (11)	C5—C6—H6	118.3
O13W—Pr1—O8^ii^	120.15 (9)	O9—C16—Pr2^v^	64.3 (2)
O13W—Pr1—N1	84.09 (11)	O9—C16—C17	120.9 (3)
O13W—Pr1—C15^ii^	95.07 (11)	O10—C16—Pr2^v^	56.0 (2)
C15^ii^—Pr1—Pr1^i^	58.24 (8)	O10—C16—O9	120.3 (4)
O12^iv^—Pr2—O9^v^	78.00 (8)	O10—C16—C17	118.6 (3)
O12^iv^—Pr2—O4	79.32 (8)	C17—C16—Pr2^v^	172.4 (2)
O12^iv^—Pr2—O3	129.85 (8)	N1—C2—C1	114.8 (3)
O12^iv^—Pr2—O11^iv^	51.70 (9)	N1—C2—C3	122.7 (3)
O12^iv^—Pr2—C23^iv^	26.01 (9)	C3—C2—C1	122.5 (3)
O12^iv^—Pr2—C16^v^	83.43 (9)	O5—C8—C9	115.7 (3)
O15W—Pr2—O12^iv^	123.55 (9)	O6—C8—O5	126.7 (3)
O15W—Pr2—O9^v^	138.66 (9)	O6—C8—C9	117.5 (3)
O15W—Pr2—O9	84.33 (10)	C6—C5—C7	119.8 (3)
O15W—Pr2—O4	102.48 (10)	C4—C5—C6	118.1 (3)
O15W—Pr2—O10^v^	145.79 (9)	C4—C5—C7	122.1 (3)
O15W—Pr2—O3	78.21 (10)	O2—C1—O1	124.8 (3)
O15W—Pr2—O14W	75.74 (9)	O2—C1—C2	118.6 (3)
O15W—Pr2—O11^iv^	73.99 (9)	O1—C1—C2	116.5 (3)
O15W—Pr2—C23^iv^	98.70 (10)	C9—C14—C15	123.4 (3)
O15W—Pr2—C16^v^	150.18 (9)	C13—C14—C15	117.1 (3)
O9—Pr2—O12^iv^	74.68 (8)	C13—C14—C9	119.3 (3)
O9—Pr2—O9^v^	66.86 (9)	O4—C7—C5	117.2 (3)
O9—Pr2—O4	152.26 (8)	O3—C7—O4	121.4 (3)
O9—Pr2—O10^v^	116.47 (9)	O3—C7—C5	121.4 (3)
O9—Pr2—O3	155.19 (8)	C17—C18—H18	119.5
O9—Pr2—O11^iv^	81.57 (9)	C17—C18—C19	121.1 (4)
O9^v^—Pr2—C23^iv^	102.54 (10)	C19—C18—H18	119.5
O9—Pr2—C23^iv^	76.84 (9)	C22—C21—H21	119.6
O9—Pr2—C16^v^	92.12 (10)	C20—C21—C22	120.9 (4)
O9^v^—Pr2—C16^v^	25.27 (8)	C20—C21—H21	119.6
O4—Pr2—O9^v^	116.93 (9)	C5—C4—H4	120.4
O4—Pr2—O3	50.80 (8)	C5—C4—C3	119.2 (3)
O4—Pr2—O11^iv^	74.77 (9)	C3—C4—H4	120.4
O4—Pr2—C23^iv^	75.56 (9)	C10—C11—H11	120.0
O4—Pr2—C16^v^	94.37 (10)	C10—C11—C12	120.0 (4)
O10^v^—Pr2—O12^iv^	89.35 (8)	C12—C11—H11	120.0
O10^v^—Pr2—O9^v^	49.64 (8)	C2—C3—C4	119.0 (3)
O10^v^—Pr2—O4	72.22 (9)	C2—C3—H3	120.5
O10^v^—Pr2—O3	72.59 (9)	C4—C3—H3	120.5
O10^v^—Pr2—O11^iv^	132.78 (8)	C11—C10—H10	119.7
O10^v^—Pr2—C23^iv^	111.90 (10)	C11—C10—C9	120.5 (4)
O10^v^—Pr2—C16^v^	24.37 (8)	C9—C10—H10	119.7
O3—Pr2—O9^v^	116.93 (9)	C14—C9—C8	121.8 (3)
O3—Pr2—C23^iv^	122.92 (10)	C10—C9—C8	118.6 (3)
O3—Pr2—C16^v^	94.34 (10)	C10—C9—C14	119.5 (3)
O14W—Pr2—O12^iv^	143.58 (9)	C14—C13—H13	119.2
O14W—Pr2—O9^v^	69.69 (8)	C14—C13—C12	121.5 (4)
O14W—Pr2—O9	77.46 (8)	C12—C13—H13	119.2
O14W—Pr2—O4	130.24 (8)	C11—C12—H12	120.4
O14W—Pr2—O10^v^	82.48 (9)	C13—C12—C11	119.3 (3)
O14W—Pr2—O3	81.23 (9)	C13—C12—H12	120.4
O14W—Pr2—O11^iv^	144.54 (9)	C18—C19—H19	120.2
O14W—Pr2—C23^iv^	154.13 (9)	C20—C19—C18	119.6 (4)
O14W—Pr2—C16^v^	74.59 (9)	C20—C19—H19	120.2
O11^iv^—Pr2—O9^v^	126.43 (8)	C21—C20—H20	120.0
O11^iv^—Pr2—O3	109.85 (9)	C19—C20—C21	120.0 (4)
O11^iv^—Pr2—C23^iv^	25.69 (10)	C19—C20—H20	120.0
O11^iv^—Pr2—C16^v^	134.86 (9)		
			
Pr1^ii^—O7—C15—O8	10.0 (3)	N1—C2—C1—O1	−3.9 (5)
Pr1^ii^—O7—C15—C14	−163.8 (2)	N1—C2—C3—C4	−2.2 (6)
Pr1^iv^—O2—C1—O1	46.5 (5)	C15—C14—C9—C8	11.8 (6)
Pr1^iv^—O2—C1—C2	−131.6 (3)	C15—C14—C9—C10	−172.9 (4)
Pr1^i^—O5—C8—O6	3.6 (6)	C15—C14—C13—C12	173.7 (4)
Pr1^i^—O5—C8—C9	179.2 (2)	C22—C17—C16—O9	−88.4 (4)
Pr1^iii^—O8—C15—Pr1^ii^	−115.2 (3)	C22—C17—C16—O10	96.6 (4)
Pr1^iii^—O8—C15—O7	−124.5 (4)	C22—C17—C18—C19	−0.3 (6)
Pr1^ii^—O8—C15—O7	−9.3 (3)	C22—C21—C20—C19	−0.9 (8)
Pr1^ii^—O8—C15—C14	164.4 (3)	C17—C22—C23—O12	−15.5 (5)
Pr1^iii^—O8—C15—C14	49.2 (5)	C17—C22—C23—O11	164.4 (4)
Pr1—O6—C8—O5	−21.5 (6)	C17—C22—C21—C20	−0.5 (6)
Pr1—O6—C8—C9	162.9 (2)	C17—C18—C19—C20	−1.1 (7)
Pr1—O1—C1—O2	174.8 (3)	C23—C22—C17—C16	5.7 (6)
Pr1—O1—C1—C2	−7.1 (5)	C23—C22—C17—C18	−177.2 (4)
Pr1—N1—C6—C5	173.0 (3)	C23—C22—C21—C20	177.9 (4)
Pr1—N1—C2—C1	11.0 (4)	C6—N1—C2—C1	−175.6 (3)
Pr1—N1—C2—C3	−171.3 (3)	C6—N1—C2—C3	2.1 (6)
Pr1^ii^—C15—C14—C9	161.5 (9)	C6—C5—C7—O4	−165.8 (4)
Pr1^ii^—C15—C14—C13	−13.1 (13)	C6—C5—C7—O3	13.7 (6)
Pr2^iii^—O12—C23—O11	−0.1 (4)	C6—C5—C4—C3	2.2 (6)
Pr2^iii^—O12—C23—C22	179.8 (3)	C16—C17—C18—C19	176.9 (4)
Pr2—O9—C16—Pr2^v^	−176.4 (6)	C2—N1—C6—C5	0.2 (6)
Pr2^v^—O9—C16—O10	1.1 (3)	C5—C4—C3—C2	0.0 (6)
Pr2—O9—C16—O10	−175.3 (4)	C1—C2—C3—C4	175.3 (4)
Pr2—O9—C16—C17	9.8 (8)	C14—C13—C12—C11	0.7 (7)
Pr2^v^—O9—C16—C17	−173.8 (3)	C7—C5—C4—C3	−175.8 (4)
Pr2—O4—C7—O3	−4.0 (4)	C18—C17—C16—O9	94.5 (4)
Pr2—O4—C7—C5	175.5 (3)	C18—C17—C16—O10	−80.5 (5)
Pr2^v^—O10—C16—O9	−1.2 (4)	C18—C19—C20—C21	1.8 (8)
Pr2^v^—O10—C16—C17	173.8 (3)	C21—C22—C17—C16	−175.8 (4)
Pr2—O3—C7—O4	3.9 (4)	C21—C22—C17—C18	1.2 (6)
Pr2—O3—C7—C5	−175.5 (3)	C21—C22—C23—O12	166.1 (4)
Pr2^iii^—O11—C23—O12	0.1 (4)	C21—C22—C23—O11	−14.0 (6)
Pr2^iii^—O11—C23—C22	−179.8 (3)	C4—C5—C7—O4	12.2 (6)
O7—C15—C14—C9	−129.3 (4)	C4—C5—C7—O3	−168.4 (4)
O7—C15—C14—C13	56.1 (5)	C11—C10—C9—C8	173.9 (4)
O5—C8—C9—C14	39.7 (5)	C11—C10—C9—C14	−1.5 (6)
O5—C8—C9—C10	−135.6 (4)	C3—C2—C1—O2	−3.4 (6)
O8—C15—C14—C9	56.9 (5)	C3—C2—C1—O1	178.4 (4)
O8—C15—C14—C13	−117.7 (4)	C10—C11—C12—C13	−0.6 (7)
O6—C8—C9—C14	−144.3 (4)	C9—C14—C13—C12	−1.2 (6)
O6—C8—C9—C10	40.4 (5)	C13—C14—C9—C8	−173.7 (4)
N1—C6—C5—C7	175.6 (4)	C13—C14—C9—C10	1.6 (6)
N1—C6—C5—C4	−2.4 (6)	C12—C11—C10—C9	1.0 (7)
N1—C2—C1—O2	174.4 (3)		

Symmetry codes: (i) −*x*+1, −*y*+1, −*z*+1; (ii) −*x*+1, −*y*+2, −*z*+1; (iii) *x*, *y*+1, *z*; (iv) *x*, *y*−1, *z*; (v) −*x*+3/2, −*y*+3/2, −*z*+1/2.

### Hydrogen-bond geometry (Å, º)

**Table:**

*D*—H···*A*	*D*—H	H···*A*	*D*···*A*	*D*—H···*A*
O13*W*—H13*A*···O11^vi^	0.85	2.57	3.003 (5)	113
O13*W*—H13*A*···O16*W*^vi^	0.85	2.26	2.881 (5)	130
O13*W*—H13*B*···O2^iii^	0.86	2.52	2.820 (4)	102
O14*W*—H14*A*···O12^vii^	0.85	1.95	2.766 (4)	160
O14*W*—H14*B*···O4^iii^	0.85	2.04	2.887 (4)	174
O14*W*—H14*B*···O10^vii^	0.85	2.58	2.912 (4)	104
O15*W*—H15*A*···O16*W*	0.85	1.75	2.595 (4)	170
O15*W*—H15*B*···O4^iii^	0.85	1.92	2.725 (3)	158
O16*W*—H16*B*···O7^viii^	0.85	2.31	2.709 (4)	109
O16*W*—H16*B*···O1^ix^	0.85	2.13	2.938 (5)	158
C11—H11···O14*W*	0.93	2.51	3.375 (5)	156

Symmetry codes: (iii) *x*, *y*+1, *z*; (vi) −*x*+3/2, *y*−1, −*z*+1; (vii) −*x*+3/2, −*y*+5/2, −*z*+1/2; (viii) *x*+1/2, −*y*+2, *z*; (ix) −*x*+3/2, *y*+1, −*z*+1.
